# Gynaecologists’ perceptions of outpatient gynaecologic and obstetric care in Germany during the COVID-19 pandemic

**DOI:** 10.1186/s12913-023-10045-1

**Published:** 2023-10-10

**Authors:** Carsten Hagenbeck, Johannes Soff, Laura Mause, Jan Hoffmann, Tim Ohnhäuser, Arno Stöcker, Janine Zöllkau, Nadine Scholten

**Affiliations:** 1 Department of Gynaecology and Obstetrics, University Hospital Düsseldorf, Moorenstraße 5, 40225 Düsseldorf, Germany; 2 Faculty of Human Sciences, Faculty of Medicine and University Hospital Cologne, Institute of Medical Sociology, Health Services Research, and Rehabilitation Science, University of Cologne, Eupener Str. 129, 50933 Köln, Germany; 3 Department of Obstetrics, University Hospital Jena, Am Klinikum 1, 07747 Jena, Germany

**Keywords:** COVID-19 pandemic, Gynaecologic care, Obstetric care, Ambulatory care

## Abstract

The measures taken to contain the COVID-19 pandemic had a major impact on society, affecting medical care as well as the utilization of medical services. We aimed to identify pandemic-related changes in gynaecologic/obstetric care through the personal experience of practitioners in the outpatient sector in Germany. Three consecutive anonymous online surveys of practising gynaecologists were conducted during the pandemic (07–09/2020, 11–12/2020 and 09–11/2021). Appointment management, medical supply and patients’ demand as well as concomitant circumstances were queried. Data from 860 (393, 262 and 205 from the first, second and third surveys, respectively) respondents were analysed. At the peak of the first COVID-19 wave, more than 50% of the gynaecologists surveyed had cancelled cancer screening appointments. There was a significant association between fear of self-infection and cancellation of cancer screening appointments (p = 0.006). An increase in domestic violence was reported by 13%, an increase in obesity by 67% and more advanced tumours due to delayed screening by 24% of respondents. Primary gynaecological oncological prevention was reduced in supply and demand during the COVID-19 pandemic, and this shortfall should be addressed in future similar situations. Prenatal care has been offered continuously since the start of the pandemic in Germany.

**Supplementary Information:**

The online version contains supplementary material available at 10.1186/s12913-023-10045-1.

## Introduction

In Germany, the outpatient sector completely covers basic medical care in the areas of prevention, diagnostics, therapy and rehabilitation. Consequences for medical care have arisen in the course of the COVID-19 pandemic, on the one hand due to the direct burden on the healthcare system caused by patients suffering from COVID-19, and on the other hand due to the social measures taken to contain the pandemic [1]. Directly, there has been a change in the range of medical services offered due to cancellations by physicians, as well as a change in the utilization of medical services on the part of patients. The range of services offered by outpatient gynaecology includes, for example, the early detection of cancer (screening), but it also includes the care of pregnant women in the context of preventive examinations. Postponing early cancer detection examinations [2], as well as suspending prenatal care [3], can lead to undetected progression of possible diseases and thus to a worse medical outcome. The non-utilization of medical consultations happens when the medical provider no longer offers or postpones the service or when it is no longer requested by the patient. Fear of SARS-CoV-2 infection has thus led to changes in utilization and supply, on the part of both patients [4] and physicians [5]. With a focus on family planning (fertility treatment and abortion), pregnancy, childbearing, and cancer screening, gynaecologic care includes distinct vulnerable areas and phases in women’s medical care that have been affected worldwide under the COVID-19 pandemic [6].

At the same time, women worldwide have suffered increased consequences of the social measures taken to contain the pandemic [7]. As an example of this, there has been an increase in domestic violence [8, 9], as well as an increase in obesity [10] and loneliness [11].

Emphasis is placed on the possible relationship between appointment cancellation and the physician’s fear of contracting SARS-CoV-2 at the beginning of the pandemic at a time when protective materials were still in short supply [12]. At the same time, the question of what consequences the pandemic will have for health care in the medium to long term is addressed, first, through changes in service utilization during the pandemic, second, through changes in health behaviour as a result of pandemic measures.

The ambulatory care sector in Germany was urged to explicitly maintain medical services open and not to postpone any important preventive measures. Likewise, appropriate measures were to be taken to minimize the risk of infection in practices and to ensure and enable infected and ill patients to receive care [13]. The aim of the analyses carried out and presented here was to investigate changes in medical care from the point of view of the outpatient sector and, explicitly, gynaecologists in private practice. In particular, gynaecologists cover the main part of cancer preventive measures for the most common carcinomas (breast and cervical carcinoma). Furthermore, pregnancy care is essential in order not to threaten safe maternal and child outcomes and to prevent avoidable harm.

## Methods

### Recruitment

Within the scope of the publicly funded study COVID-Gams (The COVID-19 Crisis and its impact on the German ambulatory sector – the physicians’ view; funded by BMBF, funding no.: BMBF 01KI2099, https://www.bmbf.de/bmbf/en), a cross-sectional, anonymous online survey focusing on the current ambulatory care was conducted using a questionnaire developed for this purpose (see supplement) at three consecutive time points (trend analysis). In addition to gynaecologists, the survey targeted family physicians, paediatricians, cardiologists, gastroenterologists and dentists. A randomly drawn sample was recruited via the physicians’ register with the help of the National Association of Statutory Health Insurance Physicians (closed survey). Therefore, 2000 gynaecologists in primary care (general practice) were invited by telefax to participate in the online survey (access via QR code). At the same time, the survey was advertised via the professional association, and participation was called for (open survey).

The first survey covered the physicians’ experiences during the peak of the first COVID-19 wave in Germany (March/April 2020) and was carried out between 13 July and 14 September 2020.The second survey was conducted from 16 to 2020 to 31 December 2020 and the third survey from 14 to 2021 to 30 November 2021.

A positive vote by the Medical Ethics Committee of the University of Cologne has been obtained for the survey (20–1169_1) and covers all three surveys. The survey was conducted anonymously by obtaining implicit consent and without the collection of directly personally identifiable variables. Participation in the survey was voluntary, and no expense allowance was paid. The estimated time required for the survey was 25 min.

### Survey instruments

Across all three survey waves, the survey consisted of generic survey instruments that were directed at all addressed physician groups, as well as differentiated questions that were explicitly directed only at the specific specialists. Here, the online survey has been filtered accordingly. Due to the dynamics of the pandemic, survey instruments are self-developed and adapted according to the course of the pandemic by setting different thematic focuses within the three surveys.

#### Changes in medical care

In the first two survey waves, the gynaecologists were questioned as to whether the mentioned type of appointments was cancelled or postponed indefinitely by them. The following types of appointments were explicitly queried: early cancer detection and follow-up care, pregnancy care, fertility treatment, family planning and contraception counselling and abortions. The possible answers were 1 = this type of appointment was cancelled or postponed, 2 = this type of appointment was offered further and 3 = our practice does not offer this type of appointment in general.

The extent to which patients’ requests had changed from the gynaecologists’ perspective was queried within all three survey waves as follows: Were there more or fewer of the following requests from patients than before the outbreak of the pandemic? The following treatment occasions were queried: Early cancer detection and follow-up care, pregnancy care, fertility treatment, family planning and contraception counselling and abortions. The possible answers were 1 = much more, 2 = little more, 3 = same, 4 = little less and 5 = much less.

#### Provider and practice-specific characteristics and socio-demographic factors

An item included in the survey in the first wave was concern about becoming infected oneself. This was queried as follows: How worried were you that you might become infected yourself? The response categories were as follows: 1 = very high, 2 = rather high, 3 = rather low, 4 = very low and 5 = not specified. The following data were also included in the group comparisons/multivariate analyses: Age of provider in categories: under 30, 31–40, 41–50, 51–60 and over 60 years), gender (male, female, diverse), size of the town in which the practice is located (under 5.000, > 5.000–20.000, > 20.000–100.000, > 100.000 inhabitants).

#### Impact of the pandemic on women’s health and social consequences

The impact of the COVID-19 pandemic, as well as the measures taken in its response on women’s health, was queried in the following dimensions: What social consequences did you see or fear? Increase in domestic violence (survey waves 2 and 3), effects on the desire to have children (survey waves 2 and 3), increase in advanced carcinomas due to the cancellation of screening and diagnostics (survey wave 3) and increase of obesity (survey wave 3) and increase in psychological stress (survey waves 2 and 3) and social inequality (survey wave 3). The given answer categories were ‘seen’, ‘feared’ or ‘neither’. With regard to the question about the increase in obesity, the survey also asked whether patients from deprived social strata were particularly affected by this (response categories: yes, no). All the information given here is based on the personal perception of the responding gynaecologist and not on objective measurements.

The survey was created and conducted in LimeSurvey. Group differences regarding fear of self-infection and cancellation of screening appointments were calculated using the two-sample Wilcoxon rank-sum (Mann-Whitney) test, which is suitable for investigating group differences in independent samples even if the normal distribution assumption is violated.

Data preparation and statistical analyses were performed using STATA 15 [14] and R 4.2.2 [15] with the Integrated Development Environment R Studio [16] (Version 2022.12.0 + 353) and further software packages [17–25].

## Results

For the first survey, the data on 1703 physicians in private practice could be analysed, of which 393 (23.1%) were gynaecologists. For the second survey, this figure was 262 (14.7%) gynaecologists from a total of 1782 physicians in private practice, and for the third survey, 205 (18.3%) gynaecologists from 1122 physicians in private practice. Characteristics of the surveyed gynaecologists are presented in Table 1. Table one shows the descriptive data, consisting of absolute numbers of cases and percentages, including missing values (unknown). All data refer to the open and closed survey.

**Table 1:** Characteristics of the surveyed gynaecologists

	1st survey wave	2nd survey wave	3rd survey wave
Characteristic	n = 393	n = 262	n = 205
Self-employed in the practice, n (%)	352 (90.0)	241 (93.1)	184 (92.5)
Unknown	2	3	6
Working in a solo practice, n (%)	221 (56.8)	160 (61.3)	134 (66.3)
Unknown	4	1	3
Number of physicians working in the practice (respondent included), n (%)			
1	161 (42.5)	123 (47.7)	
3	112 (29.6)	75 (29.1)	28 (42.4)
2	57 (15.0)	24 (9.3)	13 (19.7)
4	20 (5.3)	17 (6.6)	10 (15.2)
5 or more	29 (7.7)	19 (7.4)	15 (22.7)
Unknown	14	4	139
Size of the town in which the practice is located, n (%)			
Rural community (< 5,000 inhabitants)	9 (2.3)	7 (2.7)	7 (3.5)
Small town (> 5,000–20,000 inhabitants)	93 (24.0)	55 (21.2)	49 (24.3)
Medium-sized town (> 20,000–100,000 inhabitants)	111 (28.6)	82 (31.5)	48 (23.8)
Large city (> 100,000 inhabitants)	175 (45.1)	116 (44.6)	98 (48.5)
Unknown	5	2	3
Age, n (%)			
31 to 40 years	12 (3.1)	8 (3.1)	8 (3.9)
41 to 50 years	96 (24.6)	66 (25.3)	48 (23.6)
51 to 60 years	199 (51.0)	132 (50.6)	108 (53.2)
over 60 years	83 (21.3)	55 (21.1)	39 (19.2)
Unknown	3	1	2
Number of years worked in the outpatient sector, Mean (SD)	17 (9.1)	17 (8.4)	17 (8.9)
Unknown	6	3	3
Gender, n (%)			
male	78 (20.1)	57 (21.8)	51 (25.1)
female	311 (79.9)	205 (78.2)	150 (73.9)
diverse			2 (1.0)
Unknown	4		2
Fear of self-infection, n (%)			
Very high	59 (15.4)	14 (5.4)	
Quite high	113 (29.5)	102 (39.2)	
Quite low	171 (44.6)	116 (44.6)	
Very low	40 (10.4)	28 (10.8)	
Unknown	10	2	

### Supply of and demand for outpatient gynaecological services

**Fig. 1:**
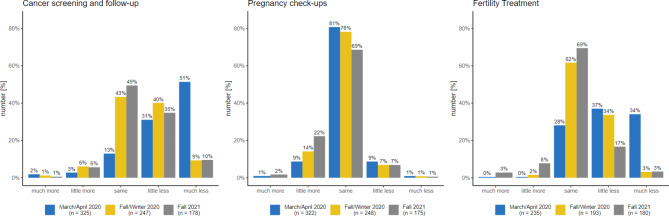
Perceived demand for services by patients over the course of the pandemic

At the time of the peak of the first COVID-19 wave in Germany (March/April 2020), more than 50% of the gynaecologists surveyed had cancelled cancer screening appointments on the doctor’s side/practice side. In fall/winter 2020, on the other hand, only 1% of those surveyed said that they did not offer cancer screening appointments at the time. However, nearly 30% of gynaecologists reported that cancer screenings were requested slightly less often, and 6% reported that they were requested much less often by patients at that time (winter 2020) (see Fig. 1). Pregnancy check-ups, on the other hand, were not cancelled by physicians during the pandemic. A small decrease in demand for pregnancy check-ups was reported by 8.7% of gynaecologists surveyed regarding March/April 2020. By contrast, 54% of the gynaecologists surveyed cancelled or indefinitely postponed fertility treatments during the same period. Again, regular fertility treatments appear to be resuming in the fall/winter, with fewer than 2% of gynaecologists surveyed stating that such treatments are still not being performed. Respondents view the demand for fertility treatments as having largely returned to pre-pandemic levels by autumn 2021. In all, 86% of the gynaecologists surveyed (first survey wave) do not offer abortions on principle. Of those who do, 98% still did so during the first wave of the pandemic.

Based on their subjective self-assessment, 45% of gynaecologists questioned confirmed that cancellations of scheduled appointments by the patients themselves had negative consequences for the state of health of these patients during the first wave of the pandemic.

### Gynaecologists’ concerns about becoming infected

Concern about becoming infected with SARS-CoV-2 varied widely among practicing gynaecologists in March/April 2020, with 15% reporting having very high worries, 30% being rather worried, 45% having rather low worries and 10% having very low worries. There was no correlation between age or gender and worries about becoming infected. Examining the gynaecologists who are self-employed and work alone in their practice (n = 131), the Wilcoxon rank sum test (p = 0.006) showed a significant difference in fear of self-infection between physicians who cancelled cancer screening appointments and those who continued to offer them. If the infection concern variable was entered dichotomously (fear of self-infection yes/no) into logistic regression model, we found that the likelihood of cancelling cancer screening appointments tripled if the physician was concerned about becoming infected themself (Table 2). For the cancellation of fertility appointments, no significant association of infection fear could be shown, and prenatal care appointments were not cancelled.

**Table 2:** Association between fear of self-infection and continued to offer early cancer detection and follow-up care (n = 128), multivariate analysis

Characteristic^1^	OR (95% CI)^1,2^	p-value^1^
Fear of self-infection (Ref. No)		
Yes	3.02 (1.47 to 6.36)	0.003
Age	0.97 (0.60 to 1.59)	0.91
Gender (Ref. Male)		
Female	0.63 (0.25 to 1.54)	0.32

^1^Tjur’s R² = 0.076

^2^OR = Odds Ratio, CI = Confidence Interval

### Impact of the pandemic on women’s life circumstances

An increase in domestic violence was feared by 81% and actually seen by 8% of the gynaecologists surveyed at the time of the second wave of the survey. At the time of the third wave of the survey, the percentage of those who actually saw an increase in domestic violence had increased to 13%, with an additional 81% fearing this.

**Fig. 2:**
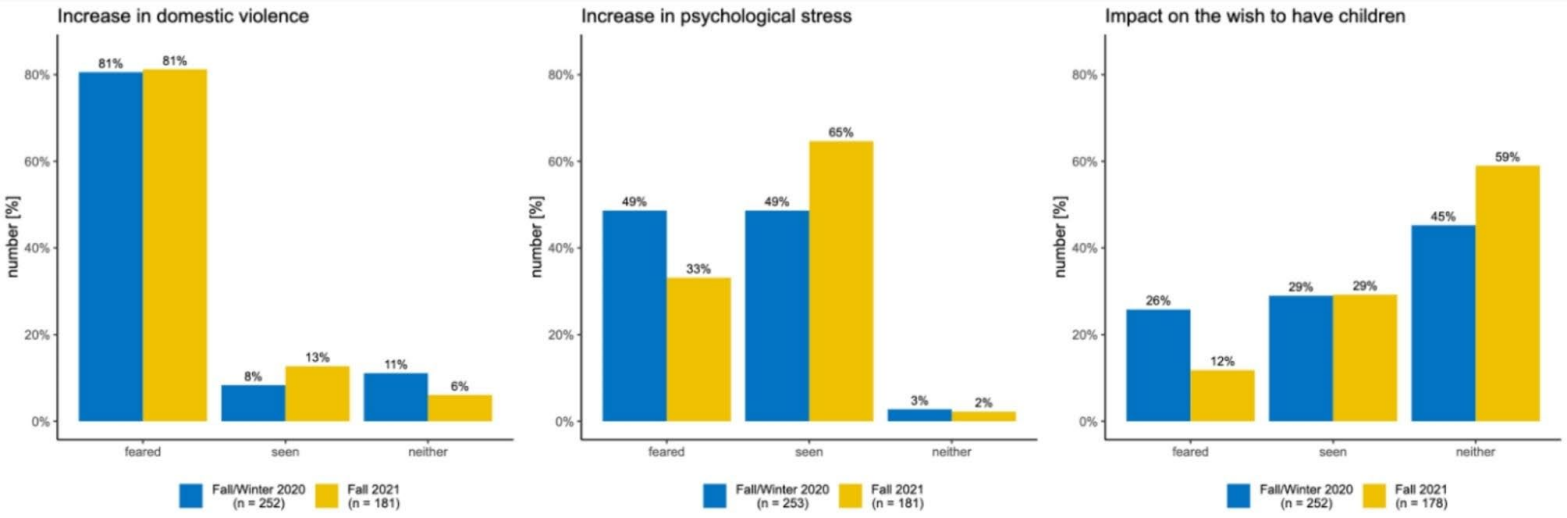
Assessment of the consequences of the pandemic from the point of view of the responding gynaecologists

With regard to the increase in psychological stress, the proportion of those who saw it increased from 49 to 65% from the second to the third survey wave. The impact of the pandemic on a possible desire to have children was seen consistently across both survey waves in about 30% of respondents (see Fig. 2). The increase in advanced carcinomas due to the cancellation of screening appointments was feared by nearly 50% and confirmed by 24% of gynaecologists in fall 2021.

A statistical association between the size of the town in which the practice is located and the assessment of the consequences of the pandemic was not shown for the increase in domestic violence, nor the increase in advanced carcinomas. An increase in social inequality in the fall of 2021 was feared by 52% and actually seen by 36% of responding gynaecologists. The increase in obesity was seen by 67% at this time, with 61% of gynaecologists stating that this particularly affected socially deprived patients.

## Discussion

The study aimed to analyse pandemic-related changes in gynaecological and obstetric care in the outpatient sector, changes in women’s life circumstances and medical care, provided by private practitioners and as perceived from their perspectives. Overall, a variety of changes in the field of outpatient gynaecological and obstetric care are evident in the wake of the COVID-19 pandemic in Germany. These include changes not only in the range of services offered but also in the perceived utilization of services by patients.

Shown here, especially in the first wave of the COVID-19 pandemic in spring 2020 in Germany, was impeded access to cancer screening and follow-up services due to provider cancellations. Our results are in line with findings from other countries. Mayo et al. showed in their review that the performance of cancer screening examinations decreased significantly across the different indications during the pandemic. In the field of gynaecology, breast cancer screening and cervical screening were explicitly investigated, and a significant decline in utilization was detected [26]. In Germany, palpation of the breast by the gynaecologist is recommended every year from the age of 30 on [27] which is particularly relevant as there is evidence that professional palpation of the mamma leads to cancer being found at an earlier stage, which in turn may also have an impact on mortality [28]. With regard to the mammography screening programme, which in Germany is a screening programme independent of outpatient gynaecological care, there has been a significant decline in utilisation [29]. Thus, especially for the diagnosis and therapy of breast cancer, the consequences of suspending mammography must be seen and discussed separately from cancer screening via palpation and eventually subsequent mammography. We observed a significant association between the provider’s cancellation of cancer screening appointments and his/her fear about contracting COVID-19 him/herself. Overall, about 45% of the gynaecologists surveyed were concerned about becoming infected at the time of the first survey, which in turn had a concrete negative impact on the care they provided. Medical personnel’s concern about becoming infected was examined in a wide range of settings during the pandemic [30]. The likelihood of cancelling an appointment triples when there is fear of infection among caregivers. The negative synergism of both the limited screening services offered by physicians and the non-utilisation by patients, as well as the resulting reduction in primary oncological prevention, suggest a dramatic effect on impending oncological incidences. Based on administrative data, a statistically and clinically relevant decrease in the number of cervical carcinoma screening examinations was observed in Germany, with women aged 20–34 being particularly affected [31]. For the U.S., a 94% decrease in cervical carcinoma screening and a 35% decrease compared to the pre-pandemic period were recorded, and there were concerns about marginalized groups being particularly affected [32]. Delaying cancer screening can lead to later detection of an existing tumour and thus to a delayed start of therapy for a more advanced tumour, resulting in a worse prognosis. 24% of gynaecologists we surveyed reported seeing more advanced tumours in the fall of 2021 than before the pandemic. For England, an increase in breast cancer deaths of 7.9–9.6% in the 5 years after diagnosis was modelled [33]. There was substantial concern about advanced oncological conditions due to the (partly self-inflicted) gap in medical services, which was in fact confirmed by 24% of our respondents.

Mainly cancer screening appointments were cancelled or postponed in the first wave of the pandemic in March/April 2020. With regard to other aspects of gynaecological outpatient services, such as prenatal care or termination of pregnancy, we observed that only a few cases of antenatal care or termination of pregnancy were affected. This leads to the assumption that only those appointments of immediate medical relevance were not affected. Here, the harm occurs in the short term, whereas if cancer prevention is postponed or suspended, the harm becomes apparent in the medium to long term.

A distinction must be made between a decline in supply due to provider cancellations and a decline in demand from patients. Our survey showed that practice-side cancellations occurred primarily in the first wave of the pandemic, but the decrease in demand was still recorded in the fall of 2021. Thus, at this point, more efforts are needed to motivate the population to resume cancer screening examinations. By actively reaching out, as an established approach, medical practices could contact and motivate their patients in the course of the pandemic and beyond.

As a concomitant finding in medical gynaecological care, we found that weight gain was observed remarkably in more than two-thirds of patients, and the lower socio-economic groups of society were identified as being particularly affected. There is substantial evidence that stress and unhealthy eating behaviours are related [34]. The influence of stress on eating behaviour has been amplified throughout the pandemic, particularly during lockdown times [35, 36]. We demonstrated through our analysis that there was even a further increase in psychological stress from the second to the third wave. Women who experience financial stress, such as having trouble paying their expenses, are 37% less likely to perform physical exercise and 32% more likely to eat unhealthy food [37], which, at the same time, further exacerbates social inequality. Accordingly, this was not unexpected, as a link between stressful events, particularly those that undermine economic stability, and changes in health behaviours, including unhealthy diets and low levels of physical activity, had already been described. However, the high number affected was unanticipated. The extent to which weight gain during the pandemic is a transient event should be investigated and, if necessary, risk factors causing such weight gain should be addressed after the pandemic has subsided, due to the possibility of long-term health damage. The aggravation of social inequality in the wake of the pandemic has been reported not only in terms of increases in obesity.

In addition to reporting on medical care-related experience, we gained further insights based on the trusting environment of a gynaecological practice and the presumption of a trusting relationship between patients and the gynaecologist. Our study reported an increase in domestic violence, which was already feared by the vast majority of gynaecologists. In Germany, as well as in Europe and worldwide, this fear could be proven in different ways (questionnaires, telephone counselling, etc.) [38–40]. We underline these mostly anonymously collected findings with our reports of physicians’ experiences.

Our findings teach us that, if similar restrictions occur in the future, oncological primary prevention, in particular, must be properly addressed, whereas obstetric care will generally remain at the same level.

### Strengths and limitations

Much of the pandemic-related research has focused on the inpatient sector. In Germany, however, the majority of medical care is provided in the outpatient sector. Through this study, it was possible to address this relevant area. In a total of three surveys, temporal trends could be identified. Due to its design (anonymous survey), the study is considered a trend analysis and not a true longitudinal study. The same physicians were invited to participate in all three surveys; however, due to the low response rate, we cannot assume that the respondents were the same. Additionally, the open survey leads to some uncertainties concerning e.g. the response rate. Due to data protection regulations, it was not possible to track how many people accessed the survey. With regard to the participants, however, we see only few missing values, so that no further selection bias can be assumed in the further course of the survey. Although the survey was anonymous, it cannot be ruled out that the physicians responded in a socially desirable manner. Since the survey was conducted in June–September 2020 retrospectively for the period March–April 2020, there is a possibility that assessments and evaluations between the observation and survey periods may have been distorted retrospectively, especially in the case of a dynamic event such as a pandemic. Another limitation is that the survey instruments are self-developed items and not validated constructs. All the results presented here do not claim to be representative, as no conclusions can be drawn in this regard due to the design. All of the results reported here are based on the self-reporting and subjective perceptions of the physicians surveyed.

## Conclusion

According to the gynecologists surveyed the supply and demand for primary oncological prevention were both reduced during the COVID-19 pandemic in Germany, especially at the beginning of the pandemic in the spring of 2020. By contrast, prenatal care has been offered more continuously, with only a slight decrease in demand. Currently, data are still lacking on whether physicians’ perceptions are also reflected in objectifiable data on utilization, as well as on the consequences of suspended cancer screening. Accompanying issues were increasing domestic violence, social inequality and, in physical terms, obesity.

### Electronic supplementary material

Below is the link to the electronic supplementary material.

Supplementary Material 1

Supplementary Material 2

## Data Availability

The datasets generated and/or analysed during the current study are not publicly available due to reasons of data protection but are available from the corresponding author on reasonable request.
