# Supplementary material for: Gynaecologists’ perceptions of outpatient gynaecologic and obstetric care in Germany during the COVID-19 pandemic
Source: BMC Health Serv Res. 2023 Oct 10;23:1079. doi: 10.1186/s12913-023-10045-1 (PMC10566176; doi:10.1186/s12913-023-10045-1)
Supplement: Supplementary file 1 — Supplementary Material 1 [file 12913_2023_10045_MOESM1_ESM.pdf]

Supplementary Table 1: Difference in fear of infection regarding cancellation/continuing cancer screening (Univariate Analysis)

Categorial

	Early cancer detection and follow-up care		
	Cancelled/postponed	Were offered further	p-value[1]
**Fear of self-infection, n**			0.006
Very high	17	8	
Quite high	20	16	
Quite low	21	35	
Very low	5	9	
**Total, n**	63	68	

[1]Wilcoxon rank sum test

Dichotomus

	Early cancer detection and follow-up care		
	Cancelled/postponed	Were offered further	p-value[1]
**Fear of self-infection, n**			0.007
Large	37	24	
Small	26	44	
**Total, n**	63	68	

[1]Wilcoxon rank sum test
