# Supplementary material for: Gynaecologists’ perceptions of outpatient gynaecologic and obstetric care in Germany during the COVID-19 pandemic
Source: BMC Health Serv Res. 2023 Oct 10;23:1079. doi: 10.1186/s12913-023-10045-1 (PMC10566176; doi:10.1186/s12913-023-10045-1)
Supplement: Supplementary file 2 — Supplementary Material 2 [file 12913_2023_10045_MOESM2_ESM.pdf]

Questionnaire

[The survey was conducted in German. You can find the English translation among the questions/statements and answers.]

Fragebogeninstrumente

Sind Sie in der Praxis selbstständig oder angestellt tätig?

Are you self-employed or employed in your practice?

- selbstständig / self-employed ☐
- angestellt / employed ☐

In welcher Praxisform arbeiten Sie?

What type of practice do they work in?

- Einzelpraxis / Single practice ☐
- Berufsausübungsgemeinschaft (früher Gemeinschaftspraxis) / Joint practice ☐

Wie viele Ärzt*innen arbeiten in der Praxis (Sie selbst mit eingeschlossen)?

How many doctors work in the practice (including yourself)?

- 1 ☐
- 2 ☐
- 3 ☐
- 4 ☐
- 5 ☐
- 6 ☐
- 7 ☐
- 8 ☐
- 9 ☐
- 10 ☐
- > 10 ☐

Wie groß ist der Ort, in dem Ihre Praxis liegt?

What is the size of the town where your practice is located?

- [ ] Landgemeinde (unter 5.000 Einwohner) < 5,000 inhabitants
- [ ] Kleinstadt (5.000 – 19.999 Einwohner) 5,000 - 19,999 inhabitants
- [ ] Mittelstadt (20.000 – 99.999 Einwohner) 20,000 - 99,999 inhabitants
- [ ] Großstadt (100.000 Einwohner oder mehr) > 100,000 inhabitants

Wie alt sind Sie?

How old are you?

- [ ] 30 Jahre oder jünger < 30 y
- [ ] 31 bis 40 Jahre 31-40 y
- [ ] 41 bis 50 Jahre 41-50 y
- [ ] 51 bis 60 Jahre 51-60 y
- [ ] über 60 Jahre > 60 y

Welchem Geschlecht fühlen Sie sich zugehörig?

What gender do you identify as?

- [ ] männlich male
- [ ] weiblich female
- [ ] divers non-binary

Seit wie vielen Jahren sind Sie im ambulanten Bereich tätig?

How many years have you been working in the outpatient field ?

Bitte tragen Sie eine Zahl ein.

[|]

Please fill in the number of years

Wie groß war Ihre Sorge davor, dass…

How concerned would you say you were...

	sehr groß	eher groß	eher gering	sehr gering	keine Angabe
… Sie sich selbst infizieren könnten? ...about getting infected?	☐	☐	☐	☐	☐
	very concerned	concerned	unconcerned	very unconcerned	no comment

Welche Art von Terminen wurden im März/April durch Ihre Praxis nicht mehr angeboten bzw. welche bestehenden Termine wurden abgesagt oder auf unbestimmte Zeit verschoben?

How many years have you been working in the outpatient field ?

Mehrfachantworten möglich

	wurden abgesagt/verschoben (cancelled / postponed)	wurden weiter angeboten (offered unchanged)	bietet unsere Praxis generell nicht an (not offered by the practice)
Krebsfrüherkennung und -nachsorge<br>Early cancer detection and follow-up care	☐	☐	☐
Schwangerschaftsvorsorge<br>Prenatal care	☐	☐	☐
Kinderwunschbehandlung<br>Fertility treatment	☐	☐	☐
Beratung zu Familienplanung und Verhütung<br>Counselling on family planning and contraception	☐	☐	☐
Schwangerschaftsabbrüche<br>Termination of pregnancy	☐	☐	☐

Welche Pandemiefolgen sehen bzw. befürchten Sie?

What effects of the pandemic do you see or fear?

	being concerned<br>befürchte ich	detected<br>habe ich gesehen	neither<br>weder noch
Zunahme häuslicher Gewalt<br>Increase in domestic violence	☐	☐	☐
Zunahme psychischer Belastungen<br>Increase in mental distress	☐	☐	☐
Zunahme der sozialen Ungleichheit<br>Increase in social inequality	☐	☐	☐
Zunahme von weit fortgeschrittenen Karzinomen aufgrund der Absage von Vorsorge- und Abklärungsterminen<br>Increase in advanced cancer findings due to appointment cancellation or postponement	☐	☐	☐
Auswirkungen auf Kinderwunsch<br>Effects on the wish to have children	☐	☐	☐

Bei welchen Personengruppen stellen Sie die Zunahme von Übergewicht fest?

In which groups of people do you notice the increase of overweight?

bei Patient*innen aus sozial schwachen Schichten ☐
in lower social classes
